# Multicountry Review of *Streptococcus pneumoniae* Serotype Distribution Among Adults With Community-Acquired Pneumonia

**DOI:** 10.1093/infdis/jiad379

**Published:** 2023-09-04

**Authors:** Lindsay R Grant, Elizabeth Begier, Christian Theilacker, Rachid Barry, Cassandra Hall-Murray, Qi Yan, Veneta Pope, Michael W Pride, Luis Jodar, Bradford D Gessner

**Affiliations:** Vaccines, Antivirals, and Evidence Generation, Pfizer Biopharma Group, Collegeville, Pennsylvania, USA; Vaccines, Antivirals, and Evidence Generation, Pfizer Biopharma Group, Dublin, Ireland; Vaccines, Antivirals, and Evidence Generation, Pfizer Pharma GmbH, Berlin, Germany; Vaccines, Antivirals, and Evidence Generation, Pfizer Biopharma Group, Collegeville, Pennsylvania, USA; Vaccines, Antivirals, and Evidence Generation, Pfizer Biopharma Group, Collegeville, Pennsylvania, USA; Vaccines, Antivirals, and Evidence Generation, Pfizer Biopharma Group, Collegeville, Pennsylvania, USA; Vaccines, Antivirals, and Evidence Generation, Pfizer Biopharma Group, Collegeville, Pennsylvania, USA; Vaccine Research and Development, Pfizer Vaccines, Pearl River, New York, USA; Vaccines, Antivirals, and Evidence Generation, Pfizer Biopharma Group, Collegeville, Pennsylvania, USA; Vaccines, Antivirals, and Evidence Generation, Pfizer Biopharma Group, Collegeville, Pennsylvania, USA

**Keywords:** adults, community-acquired pneumonia, pneumococcal vaccines, serotype distribution, *Streptococcus pneumoniae*

## Abstract

**Background:**

Nonbacteremic community-acquired pneumonia (CAP) is a leading presentation of severe pneumococcal disease in adults. Serotype-specific urinary antigen detection (UAD) assay can detect serotypes causing pneumococcal CAP, including nonbacteremic cases, and guide recommendations for use of higher valency pneumococcal conjugate vaccines (PCVs).

**Methods:**

Adult CAP serotype distribution studies that used both Pfizer UADs (UAD1, detects PCV13 serotypes; UAD2, detects PCV20 non-PCV13 serotypes plus 2, 9N, 17F, and 20) were identified by review of an internal study database and included if results were published. The percentages of all-cause radiologically confirmed CAP (RAD + CAP) due to individual or grouped (PCV13, PCV15, and PCV20) serotypes as detected from culture or UAD were reported.

**Results:**

Six studies (n = 2, United States; n = 1 each, Germany, Sweden, Spain, and Greece) were included. The percentage of RAD + CAP among adults ≥18 years with PCV13 serotypes equaled 4.6% to 12.9%, with PCV15 serotypes 5.9% to 14.5%, and with PCV20 serotypes 7.8% to 23.8%. The percentage of RAD + CAP due to PCV15 and PCV20 serotypes was 1.1–1.3 and 1.3–1.8 times higher than PCV13 serotypes, respectively.

**Conclusions:**

PCV13 serotypes remain a cause of RAD + CAP among adults even in settings with pediatric PCV use. Higher valency PCVs among adults could address an important proportion of RAD + CAP in this population.

## Teaser

Urinary antigen detection assays enhanced detection of *S pneumoniae* community-acquired pneumonia (CAP) and detected an important fraction of CAP due to serotypes covered by PCV13 and PCV20 among adults ≥18 years from the United States, Germany, Greece, Spain, and Sweden.

*Streptococcus pneumoniae* is a commonly identified cause of bacterial community-acquired pneumonia (CAP). Two types of pneumococcal vaccines—pneumococcal conjugate vaccines (PCVs) and a 23-valent pneumococcal polysaccharide vaccine (PPSV23)—are recommended in adults to prevent pneumococcal disease. Given that nonbacteremic CAP represents the most common presentation of pneumococcal disease in adults [1] and that the disease burden of non-PCV13-emerging serotypes remains substantial [2, 3], vaccine manufacturers have developed higher valency PCVs to expand coverage to new serotypes. PCV15 (including PCV13 serotypes [1, 3, 4, 5, 6A, 6B, 7F, 9V, 14, 18C, 19A, 19F, and 23F] plus 2 additional serotypes: 22F and 33F [Merck & Co., Inc., Kenilworth, NJ]) and PCV20 (including PCV13 serotypes plus 7 additional serotypes: 8, 10A, 11A, 12F, 15B, 22F, and 33F [Pfizer Inc., New York, NY]) have been recently approved by the US Food and Drug Administration and European Medicines Agency for use in adults aged ≥18 years [4–6].

Diagnostic tests that detect pneumococcal carbohydrate antigens in the urine have multiple advantages over culture-based methods, because they are less affected by antimicrobial treatment, and sample collection is relatively easy and noninvasive. Currently, 2 urine-based antigen testing platforms exist: the pan-serotype pneumococcal urinary antigen test (PUAT) and the serotype-specific urinary antigen detection (UAD) assays. The PUAT does not distinguish between pneumococcal serotypes. Pfizer (which manufactures PCV13 and PCV20), Merck (which manufactures PCV15), and the United Kingdom (UK) Health Protection Agency have each developed proprietary serotype-specific UAD assays [7–11] to overcome the limitations associated with traditional pneumococcal detection methods (culture or PUAT). The Pfizer and UK UAD assays both detect serotypes in PPSV23 plus serotype 6A. The Merck UAD detects the PCV15 serotypes only. Both the Pfizer and Merck UAD assays have been clinically validated, although different methods were used for the determination of diagnostic positivity cut-off limits. The Pfizer and UK UAD assays have also demonstrated high sensitivity and specificity using a gold standard of blood culture-positive bacteremic CAP [7–9]. The sensitivity and specificity of UAD assays against nonbacteremic CAP has not been determined due to the lack of a comparative gold standard.

Several prospective CAP studies have used the UAD assays to examine *S pneumoniae* serotype distribution in adults; all but 1 has used the Pfizer UAD assays (1 study used the UK UAD [2]). In this review, we summarize *S pneumoniae* serotype distribution in adults with bacteremic and nonbacteremic CAP, using data from the Pfizer UAD studies only, which were conducted using the same methodology and analytic approach.

## METHODS

### Study Selection

We reviewed an internal database of studies to identify those eligible for inclusion based on the following criteria: prospective enrollment of adults ≥18 years of age hospitalized with CAP, use of both the Pfizer UAD1 (detects the PCV13 serotypes plus 6C) and UAD2 (detects 11 additional serotypes: 2, 8, 9N, 10A, 11A, 12F, 15B/15C, 17F, 20, 22F, and 33F) assays to test urine samples, and publication of results as a manuscript or conference presentation/poster before May 2021.

The Pfizer serotype-specific UAD is available only for research and requires the involvement of Pfizer staff to conduct the analysis. Because all studies were known to Pfizer staff, no systematic search was conducted and thus PRISMA guidelines did not apply. Because we included only published studies, additional ethical review or informed consent was not sought or obtained.

### Data Extraction and Analysis

Studies that met the eligibility criteria were included in the analysis. Study characteristics obtained for each study included study period, patient inclusion/exclusion criteria, and specimen type and testing methods for *S pneumoniae* detection. We also extracted the following patient demographics and baseline characteristics as reported for participants in each publication: comorbidities, vaccination history (pneumococcal and flu vaccines), CAP severity on admission as measured by the pneumonia severity index (PSI) or the CURB-65 score, and CAP clinical outcomes (eg, in-hospital mortality). Because the German data publication did not include participant characteristics or individual serotype contribution, these data were requested from the corresponding author to align with those available from other countries.

Data on *S pneumoniae* detection as determined by culture, BinaxNow *S pneumoniae* test (a commercially available PUAT for *S pneumoniae* detection [Abbot Diagnostics Scarborough Inc., Scarborough, ME]) and UAD assays were extracted and tabulated. *S pneumoniae* detection enhancement with the use of UAD assays or BinaxNow was calculated and plotted. The fold change in *S pneumoniae* detection was estimated in 2 ways. The first fold-change calculations quantified the unique contribution to *S pneumoniae* detection by urine-based assays, specifically UAD versus culture + BinaxNow or BinaxNow versus culture + UAD. The second quantified the benefit of urine-based *S pneumoniae* detection assays compared with culture.

Serotype distribution as determined by culture and UAD assays was also extracted. Individual serotypes were grouped as PCV serotypes (PCV7, PCV13, PCV15, and PCV20) or non-PCV serotypes and presented as counts and proportions of the total number of CAP study participants eligible for inclusion in the analytic population. Serotype distribution data were stratified by age group if available. The relative increase in the percentage of all-cause CAP covered by higher valency PCVs (PCV15 and PCV20) compared with PCV13 was calculated.

To aid the interpretation of the serotype distribution data, pneumococcal vaccination policies for children and adults were extracted from national recommendations and summarized. Vaccination policies on pediatric PCV product and schedule, definition of age and risk groups, definition of comorbidities, and recommendation and reimbursement status in adults for PCV13 and PPSV23 were presented.

## RESULTS

### Study Characteristics

Six studies met the study eligibility criteria and were included in the analysis [3, 12–16], including studies from the United States (n = 2), Germany, Sweden, Spain, and Greece (n = 1 each) (Table 1).

**Table 1.** Summary of Design and Methods Used in Prospective Adult CAP Serotype Distribution Studies

Study Characteristics	United States (General Population)	United States (Native American Population)	Germany	Sweden	Spain^a^	Greece
Study period	Oct 2013–Sept 2016	Mar 2016–Mar 2018	Jan 2013–Dec 2019	Sept 2016–Sept 2018	Nov 2011–Nov 2018	Nov 2017–Apr 2019
Inclusion Criteria						
≥18 years of age	✓	✓	✓	✓	✓	✓^b^
Hospitalized/medically attended	✓	✓	✓	✓	✓	✓
Signs or symptoms of pneumonia	✓	✓	✓	✓	✓	✓
Radiographic finding consistent with pneumonia^c^	✓	…	✓	✓	✓	…
Exclusion Criteria						
Hospital-acquired pneumonia	✓	✓	✓	✓	✓	✓
Previous enrollment	✓	…	…	…	…	…
Clear alternative diagnosis	…	✓	✓	…	…	…
Immunocompromised	…	…	✓	…	✓	…
Pneumonia is terminal event	…	…	✓	…	…	…
Radiologically confirmed CAP analysis population	✓	✓	✓	✓	✓	✓
Specimen Collection						
Blood	✓	✓	…	✓	✓	✓
Lower respiratory tract	✓	…	…	…	✓	✓
Urine	✓	✓	✓	✓	✓	✓
Specimen Testing						
Culture of blood or lower respiratory tract specimens	✓	✓	…	✓	✓	✓
Serotyping cultured isolates^d^	✓	✓	…	✓	✓	✓
BinaxNOW *Streptococcus pneumoniae* test^e^	✓	✓	…	✓	✓	✓
Serotype-specific urinary antigen detection assays (UAD1/UAD2)^f^	✓	✓	✓	✓	✓	✓
Study reference	[14]	[15]	[12]	[13]	[3]	[16]

Abbreviations: Apr, April; CAP, community-acquired pneumonia; Jan, January; Mar, March; Nov, November; Oct, October; Sept, September; UAD, urinary antigen detection.

^a^The study was conducted in Catalonia, Valencia, Basque country, and Galicia regions of Spain.

^b^The age group targeted for inclusion in Greece was ≥19 years of age.

^c^The interpretation of the chest radiograph and determination of whether radiographic findings were suggestive of pneumonia were made by a certified radiologist.

^d^Serotyping methods include Quellung reaction, multiplex polymerase chain reaction, and latex agglutination.

^e^The BinaxNOW *S pneumoniae* test is a pneumococcal urinary antigen detection test that provides qualitative detection of *S pneumoniae* from urine.

^f^The UAD assays (UAD1 and UAD2) use validated Luminex technology to detect 24 serotypes including 1, 3, 4, 5, 6A/6C, 6B, 7F, 9V, 14, 18C, 19A, 19F, and 23F detected by UAD1 and 2, 8, 9N, 10A, 11A, 12F, 15B/15C, 17F, 20, 22F, and 33F detected by UAD2.

Patient inclusion criteria across the 6 studies were adult patients ≥18 or ≥19 years of age hospitalized with signs or symptoms of pneumonia; the definition and the number of presenting symptoms varied across the studies (Supplementary Table 1). All studies excluded hospital-acquired pneumonia. Studies from Germany and Spain also excluded immunocompromised patients, whereas the others did not. The analytic population across all studies included adult patients hospitalized with radiographically confirmed CAP (RAD + CAP).

Five studies reported the collection of blood or lower respiratory tract cultures for *S pneumoniae* identification and serotyping (Table 1). The percentage of RAD + CAP cases with standard-of-care cultures ranged from 68.1% (Greece) to 94.4% (United States, general population). Although Germany study participants may have had standard-of-care cultures collected, the investigators did not incorporate these results in the analysis. In all studies, urine samples were collected from participants and tested by both BinaxNow and UAD assays to identify *S pneumoniae,* except for Germany, which only used the UAD assays.

### Patient Demographics and Characteristics

The number of enrolled patients with CAP ranged from N = 521 (Greece) to N = 15 572 (United States, general population) (Table 2). The analytic population of patients with RAD + CAP ranged from N = 482 (Greece) to N = 12 055 (United States, general population). The mean age of CAP patients was 61.4–70.5 years across the studies; adults ≥65 years of age accounted for 52.7% to 68.0% of the analytic population. A high proportion of patients had at least 1 comorbidity (range, from 77.3% in Germany to 94.5% among US Native Americans), and the most common comorbidities were diabetes (n = 3 studies), chronic lung disease (n = 2 studies), or chronic heart disease (n = 1 study). Disease severity was assessed by PSI grade (n = 4 studies) or CURB-65/CRB-65 (n = 4 studies). For studies that reported PSI grade, the proportion of patients that had PSI grade IV or V indicating severe disease ranged from 40.6% (Spain) to 53.5% (Greece). In-hospital mortality ranged from 2.6% to 7.1%.

**Table 2.** Demographic and Clinical Characteristics of Study Participants

Characteristics	United States (General Population)	United States (Native American Population)	Germany^a^	Sweden	Spain	Greece
Study Population Characteristics						
Total enrolled	15 572	767	1831	567	3107	521
Total RAD + CAP, overall age group, n	≥18 years: 12 055	≥18 years: 580	≥18 years: 1343	≥18 years: 518	≥18 years: 3107	≥19 years: 482
Analytic age group 1, n (%)	18–64 years: 5708 (47.3)	18–64 years: 275 (47.4)	18–59 years: 316 (23.5)	18–64 years: 169 (32.6)	18–64 years: 1164 (37.5)	19–64 years: 154 (32.0)
Analytic age group 2, n (%)	≥65 years: 6347 (52.7)	≥65 years: 305 (63.3)	≥60 years: 792 (59.0)	≥65 years: 349 (67.4)	≥65 years: 1943 (62.5)	≥65 years: 328 (68.0)
Mean age in years (SD)	64.1 (16.6)	66 (52, 79)^b^	61.4 (17.1)	69.0 (17.5)	66.8 (17.2)	70.5 (18.4)
Male sex, n/N (%)	5967/12 055 (49.5)	276/580 (47.6)	829/1343 (61.7)	282/518 (54.4)	1283/3107 (61.5)	272/482 (56.4)
Smoking	3525/12 035 (29.3)	63/580 (10.9)	NR	97/283 (34.3)	553/3107 (17.8)	217/482 (45.0)
Alcohol use	563/12 023 (4.7)	48/580 (8.3)	NR	18/510 (3.5)	114/3107 (3.7)	NR
Study Specimen Sampling Characteristics, n/N (%)						
Standard of care specimen sampling rate, n (%)	11 382/12 055 (94.4), microbiology cultures	NR	NA	478/518 (92.2)	NR	328/482 (68.1), blood or respiratory specimens
Cultures of normally sterile sites	NR	483/580 (83.3), blood	NA	476/518 (91.2), specimen type unspecified	2120/3107 (68.2), blood 231/3107 (7.4), pleural fluid	300/482 (62.2), blood
Cultures of respiratory secretions	NR	NR	NA	32/518 (6.2), specimen type unspecified	1186/3107 (38.2), sputum	73/482 (15.1), sputum
Comorbidities, n/N (%)						
Any comorbidity	10 396/12 055 (86.2)	548/580 (94.5)	1038/1343 (77.3)	401/518 (77.4)	2704/3107 (87.0)	383/482 (79.5)
At-risk comorbidity						
Chronic lung disease	5159/12 001 (43.0) COPD	66/580 (11.4)	511/1343 (38.0)	166/517 (32.1) COPD	583/3107 (18.8) COPD	71/482 (14.7) COPD
Asthma	1757/11 998 (14.6)	NR	NR	47/518 (9.1)	271/3107 (8.7)	19/482 (3.9)
Chronic heart/cardiac disease	NR	73/580 (12.6)	662/1343 (49.3)	NR	NR	NR
Heart failure	2892/12 006 (24.1)	NR	128/1343 (9.5)	96/518 (18.5)	337/3107 (10.8)	61/482 (12.7)
Coronary artery disease	3084/12 002 (25.7)	NR	NR	135/518 (26.1)	NR	85/482 (17.6)
Chronic liver disease	698/12 003 (5.8)	NR	42/1343 (3.1)	10/518 (1.9)	104/3107 (3.3)	3/482 (0.7)
Diabetes	3437/12 005 (28.6)	258/580 (44.6)	232/1343 (17.3)	87/518 (16.8)	714/3107 (23.0)	110/482 (22.8)
High-risk comorbidity, n/N (%)^c^	4637/12 055 (38.5)	NR	NA	180/518 (34.7)	NA	71/482 (14.7)
Chronic kidney disease	1843/12 001 (15.4)	NR	150/1343 (11.2)	47/517 (9.1)	297/3107 (9.6)^d^	45/482 (9.3)
Malignant disease	NA	45/580 (12.7)	168/1343 (12.2)	NR	NR	44/482 (9.0)
Hemato-oncological	360/12 008 (3.0)	NR	25/1343 (1.9)	19/517 (3.7)	NR	NR
Solid cancer	1831/12 001 (15.3)	NR	145/1343 (10.8)	106/516 (20.5)	NR	NR
Mean hospital stay in d (SD)	7.1 (5.1)	NR	NR	8.6 (18.7)	8.8 (NR)	7.5 (7.8)
ICU admission, n/N (%)	1915/11 951 (16.0%)	NR	87/1204 (7.2)	NR	313/3107 (10.1)	NR
Severity						
CURB category^e^	NR	NR	CURB-65 Mean (SD): 1.0 (1.0)	CRB-65 Mean (SD): 0.9 (0.7)	CURB-65 score 3–5, n/N (%): 401/3107 (13.8)	CURB-65 score 3–5, n/N (%): 121/482 (25.1)
PSI Grade IV or V, n/N (%)	5881/12 055 (48.8)	NR	NR	262/518 (50.6)	1250/3107 (40.6)	258/482 (53.5)
PSI mean (SD)	89.5 (44.70)	NR	NR	92.6 (35.9)	NR	NR
Vaccination History, n/N (%)						
Source of information	NR	Medical records	Patient report	Patient report, medical records	Medical records	Patient report
PPSV23 ever	NR	461/580 (79.5)	NR	NR	376/3107 (12.1)	20/482 (4.1)
PCV13 ever	NR	269/580 (46.4)	NR	NR	43/3107 (1.4)	28/482 (5.8)
Either PPSV23 or PCV13	NR	NR	180/1343 (13.4)	56/489 (11.5)	441/3107 (14.2)	48/482 (10.0)
Seasonal influenza	NR	NR	463/1343 (34.5)	172/494 (34.8)	1547/3107 (49.8)	142/482 (29.5)
Mortality, n/N (%)						
Total	1018/12 055 (9.0)	NR	NR	45/518 (8.7)	93/3107 (3.0)	NR
In hospital	537/12 055 (4.5)	NR	NR	19/518 (3.7)	81/3107 (2.6)	34/482 (7.1)
30-day	1009/12 055 (8.4)	NR	17/1343 (1.3)	21/518 (4.1)	10/3107 (0.4)	48/482 (10.0)

Abbreviations: COPD, chronic obstructive pulmonary disease; CURB, confusion, blood nitrogen urea >19 mg/dL (>7 mmol/L), respiratory rate equal to or higher than 30/min, blood pressure (BP): systolic BP <90 mmHg or diastolic BP ≤60 mmHg; ICU, intensive care unit; IQR, interquartile range; n, numerator count; N, denominator count; NA, not applicable; NR, not reported; PCV13, 13-valent pneumococcal conjugate vaccine; PPSV23, 23-valent pneumococcal polysaccharide vaccine; PSI, Pneumonia Severity Index; RAD + CAP, radiographically confirmed community-acquiredpneumonia; SD, standard deviation.

^a^Data from Germany were requested because demographic/clinical information was not provided in the publication.

^b^Reported age for US Native American population is the median and interquartile range.

^c^High-risk conditions include asplenia, cerebrospinal fluid leak, cochlear implant, hemoglobinopathy, immunosuppression therapy, nephrotic syndrome, chronic renal failure, immunodeficiency, human immunodeficiency virus (HIV) infection, acquired immunodeficiency syndrome (AIDS), cancer (including solid tumor, multiple myeloma, and other hematologic cancer treated currently or within the past 5 years), and organ/bone marrow transplantation. Studies in Germany and Spain enrolled immunocompetent adults only.

^d^For Spain, chronic renal failure was reclassified as chronic kidney disease.

^e^For CURB category, the point range is 0–5 for CURB-65 and 0–4 for CRB-65.

### Pediatric and Adult Pneumococcal Vaccination Recommendations Relevant to Community-Acquired Pneumonia Study Period

Pneumococcal conjugate vaccine pediatric schedules vary, with the United States and Greece using PCV13 in a 3 + 1 schedule, Germany and Sweden using both PCV10 and PCV13 in a 2 + 1 schedule, and Spain using PCV13 in a 2 + 1 schedule (Supplementary Table 2). PCV13 is recommended for adults with certain chronic medical conditions in the United States, Sweden, Spain (regional), and Greece, and for adults with certain immunocompromising conditions in all countries. Furthermore, PCV13 is recommended for all older adults (≥60 or ≥65 years of age) in the United States, Spain (regional), and Greece (Supplementary Table 2). For included studies, the proportion of participants vaccinated with PCV13 ranged from 1.4% to 46.4% for PPSV23 and from 10.0% to 14.2% for PCV13.

### *Streptococcus pneumoniae* Detection and Serotype Distribution

**Figure 1.**
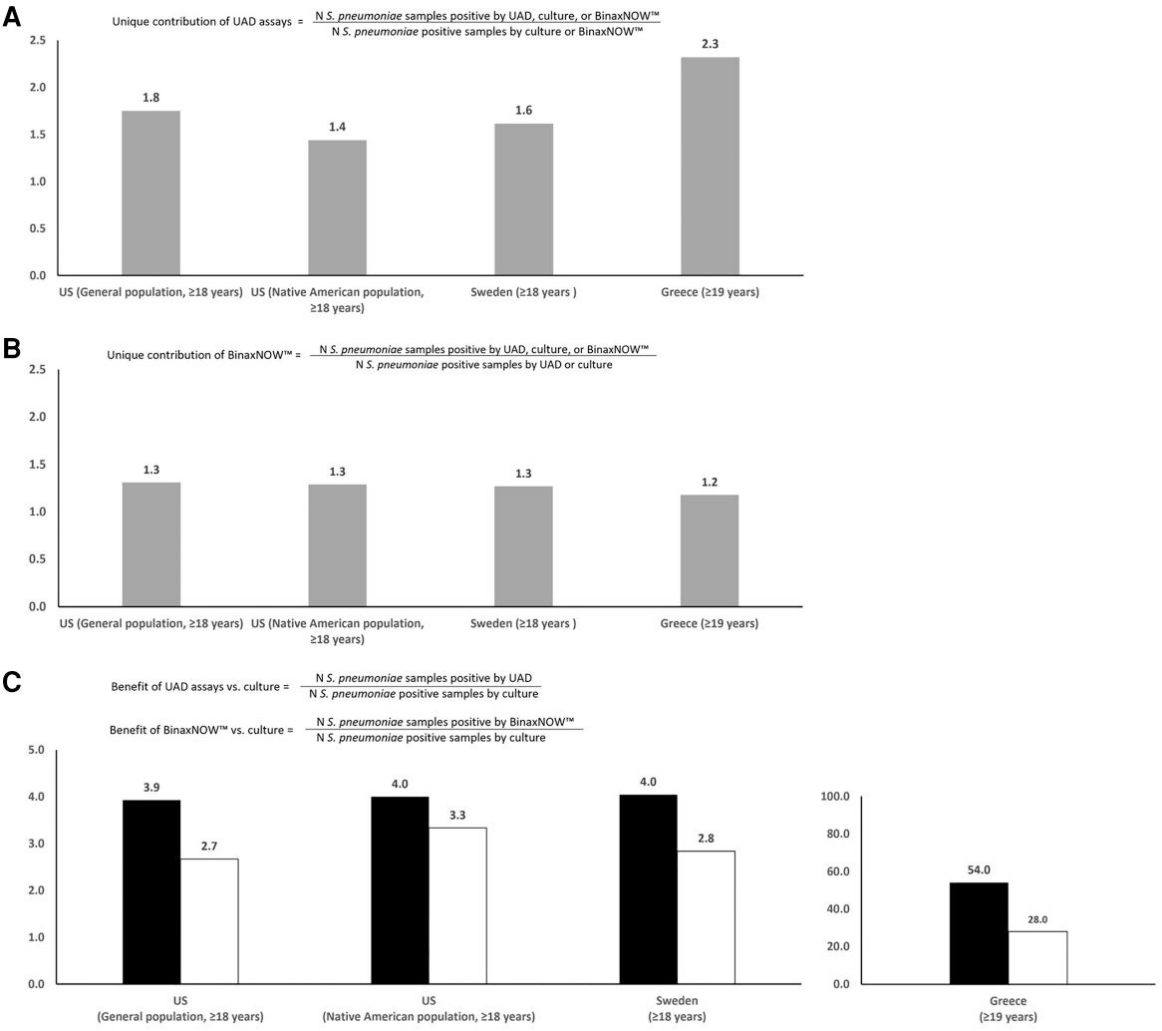
*S pneumoniae* detection increase with addition of urine-based *S pneumoniae* detection assays among participants with community-acquired pneumonia. (*A*) Streptococcus pneumoniae detection fold change to quantify unique contribution of urinary antigen detection assay (UAD) assays. (*B*) *S pneumoniae* detection fold change to quantify unique contribution of the BinaxNOW test. (*C*) *S pneumoniae* detection fold change to quantify the contribution of BinaxNOW test or UAD versus culture. In (*C*), the scale of y-axis changes for Greece compared with other countries.

Among all patients with RAD + CAP, *S pneumoniae* detected by any laboratory method ranged from 12.3% (United States, general population) to 34.0% (Spain) (Table 3). *Streptococcus pneumoniae* detection by culture or UAD assays was not available for the study conducted in Spain. Age-stratified data on *S pneumoniae* detection are shown in Supplementary Table 3. Compared with culture and BinaxNOW only, including the UAD assays as a diagnostic method increased the detection of *S pneumoniae* by 1.4 to 2.3 times across the studies (Figure 1*A*). Compared with culture and the UAD assays, including the use of the BinaxNOW increased the detection of *S pneumoniae* by 1.2 to 1.3 times (Figure 1*B*). Compared with culture, detection of *S pneumoniae* was 3.9 to 4.0 times higher for UAD and 2.7 to 3.3 times higher for BinaxNOW for 3 studies (United States, general population; United States, Native Americans; and Sweden); for Greece, the corresponding values were 54.0 times higher for UAD and 28.0 times higher for BinaxNOW (Figure 1*C*).

**Table 3.** *S pneumoniae* Detection by Laboratory Method Among Participants With RAD + CAP

Category	United States (General Population)	United States (Native American Population)	Germany	Sweden	Spain^a^	Greece
Age group, years	≥18	≥18	≥18	≥18	≥18	≥19
Total number of CAP cases	12 055	572	1343	518	1021	482
*S pneumoniae* detection by diagnostic test, n/N (%)						
Any method	1482/12 055 (12.3)	164/572 (28.6)^b^	NR	126/518 (24.3)	347/1021 (34.0)	65/482 (13.5)
Culture	262/11 382 (2.3)	29/483 (6.0)	NR	24/478 (5.0)	NR	1/328 (0.3)
UAD assay	1028/12 054 (8.5)	120/572 (21.0)	183 (13.7)^c^	97/518 (18.7)	NR	54/482 (11.2)
BinaxNOW	699/12 055 (5.8)	102/572 (17.8)	NR	68/518 (13.1)	523/3107 (16.8)^d^	28/482 (5.8)

Abbreviations: CAP, community-acquired pneumonia; n, numerator count; N, denominator count; NR, not reported; UAD, urinary antigen detection.

^a^Unless otherwise noted, data from Spain were restricted to the period of November 2016–November 2018 when UAD1 and UAD2 assays were both used.

^b^A total of 580 patients were included in the radiographically confirmed pneumonia population; of these, 572 had samples available for pneumococcal testing.

^c^Germany did not include results of standard-of-care specimens cultured for *S pneumoniae* nor was the BinaxNOW test run on urine samples.

^d^BinaxNOW test results from Spain were reported for the entire study period of November 2011–November 2018.

**Figure 2.**
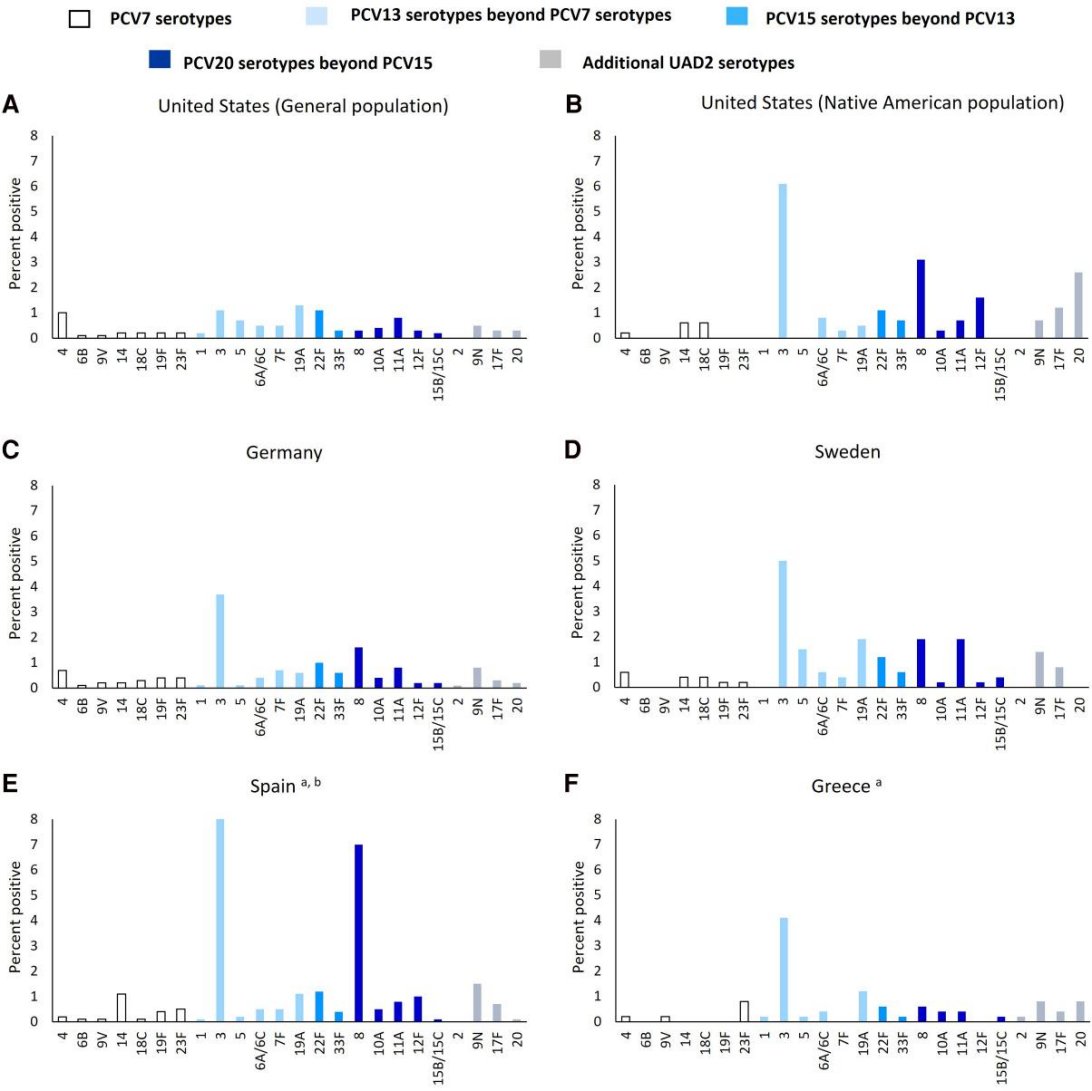
Serotype distribution as detected by culture and urinary antigen detection assay (UAD) assays among participants with community-acquired pneumonia by country. Serotype distribution data separately for bacteremic and nonbacteremic cases were not necessarily available in the original publications. PCV7, 7-valent pneumococcal conjugate vaccine; PCV13, 13-valent PCV; PCV15, 15-valent PCV; PCV20, 20-valent PCV; UAD2, UAD assay 2. (*A*) Some serotype counts from United States (Native American population), Spain, and Greece were estimated using WebPlotDigitizer, a web-based tool used to extract numerical data from figures. (*B*) Serotype counts represent data between November 2016 and November 2018 only.

Included studies were conducted after the introduction of PCVs in national pediatric National Immunization Programs (NIPs) (Supplementary Table 2). Among adults ≥18 years of age with RAD + CAP, the most common serotype detected in each study by the UAD1 or UAD2 assays was serotype 3 in 5 studies (3.7%–8.3%). Among the United States, general population, serotypes 3 and 19A were the most common at 1.1% and 1.3%, respectively, for adults ≥18 years of age with RAD + CAP (Figure 2 and Supplementary Table 4). Beyond these, serotypes 8, 9N, 11A, and 22F were also commonly detected among adults ≥18 years of age, with all but 9N covered by PCV20. Serotypes 3 and 19A remained the most common across age strata, except for Spanish patients aged 18–64 years, for whom serotype 8 was the most common (Supplementary Table 4). Serotypes detected by culture only are shown in Supplementary Table 5.

Across the studies, PCV13 serotypes accounted for 4.6% (United States, general population) to 12.9% (Spain) of all RAD + CAP cases, PCV15 serotypes accounted for 5.9% (United States, general population) to 14.5% (Spain), and PCV20 serotypes accounted for 7.8% (United States, general population) to 23.8% (Spain) as detected by UAD assays plus culture (Table 4). The relative increases of PCV15 serotype coverage over PCV13 ranged from 1.1 to 1.3 times higher, and PCV20 over PCV13 ranged from 1.3 to 2.2 times higher (Table 4). Restricting to RAD + CAP cases positive for *S pneumoniae* by any laboratory method, PCV13, PCV15, and PCV20 serotypes accounted for 34% to 54%, 43% to 60%, and 63% to 72%, respectively.

**Table 4.** Age-Stratified PCV Serotype Coverage for Adults With RAD + CAP as Determined by Culture and UAD Assays Among Participants With CAP

Category	United States (General Population)			United States (Native American Population)^a^			Germany			Sweden			Spain^a,b^			Greece^a^		
Age group, years	18–64	≥65	≥18	18–64	≥65	≥18	18–59 with CMC	≥60	≥18	18–64	≥65	≥18	18–64	≥65	≥18	19–64	≥65	≥19
Total number of RAD + CAP cases	5708	6347	12 055	274	298	572^c^	316	792	1343	169	349	518	401	620	1021	154	328	482
Coverage of grouped serotypes, n (%)																		
PCV7 serotypes^d^	71 (1.2)	53 (0.8)	124 (1.0)	NR	NR	NR	8 (2.5)	16 (2.0)	30 (2.2)	2 (1.2)	7 (2.0)	9 (1.7)	NR	NR	NR	2 (1.3)	4 (1.2)	6 (1.2)
PCV13 serotypes^e^	290 (5.1)	269 (4.2)	559 (4.6)	23 (8.4)	26 (8.7)	49 (8.6)	23 (7.3)	61 (7.7)	103 (7.7)	21 (12.4)	35 (10.0)	56 (10.8)	50 (12.5)	82 (13.2)	132 (12.9)	16 (10.4)	19 (5.8)	35 (7.3)
PCV13 serotypes not in PCV7	236 (4.1)	222 (3.5)	458 (3.8)	NR	NR	NR	15 (4.7)	45 (5.7)	73 (5.4)	20 (11.8)	28 (8.0)	49 (9.5)	NR	NR	NR	14 (9.1)	16 (4.9)	30 (6.2)
PCV15 serotypes^f^	370 (6.5)	346 (5.5)	716 (5.9)	NR	NR	NR	28 (8.9)	74 (9.4)	122 (9.1)	23 (13.6)	42 (12.0)	65 (12.5)	53 (13.2)	95 (15.3)	148 (14.5)	18 (11.7)	21 (6.4)	39 (8.1)
PCV20 serotypes^g^	497 (8.7)	441 (7.0)	938 (7.8)	NR	NR	NR	37 (11.7)	99 (12.6)	165 (12.3)	35 (20.7)	53 (15.2)	88 (17.0)	111 (27.7)	132 (21.3)	243 (23.8)	20 (13.0)	27 (8.2)	47 (9.8)
PCV20 serotypes not in PCV13	219 (3.8)	181 (2.9)	400 (3.3)	NR	NR	NR	14 (4.4)	40 (5.1)	65 (4.9)	15 (8.9)	18 (5.2)	33 (6.4)	NR	NR	NR	4 (2.6)	8 (2.4)	12 (2.5)
Serotypes detected by UAD2, but not in PCV20	69 (1.2)	54 (0.9)	123 (1.0)	NR	NR	NR	0	15 (1.9)	19 (1.4)	4 (2.4)	7 (2.0)	11 (2.1)	NR	NR	NR	3 (1.9)	6 (1.8)	9 (1.9)
Fold Increase versus PCV13																		
PCV15	1.3	1.3	1.3	–	–	–	1.2	1.2	1.2	1.1	1.2	1.2	1.1	1.2	1.1	1.1	1.1	1.1
PCV20	1.7	1.6	1.7	–	–	–	1.6	1.6	1.6	1.7	1.5	1.6	2.2	1.6	1.8	1.3	1.4	1.3

Abbreviations: CMC, chronic medical conditions; NR, not reported; PCV, pneumococcal conjugate vaccine; PCV7, 7-valent PCV; PCV13, 13-valent PCV; PCV15, 15-valent PCV; PCV20, 20-valent PCV; RAD + CAP, radiographically confirmed community-acquired pneumonia; UAD, urinary antigen detection; UAD2, UAD assay 2.

^a^Serotype counts in italics from United States (Native American population), Spain, and Greece were estimated using WebPlotDigitizer, a web-based tool used to extract numerical data from figures.

^b^Serotype counts represent data between November 2016 and November 2018 only.

^c^A total of 580 patients were included in the RAD + CAP population; of these, 572 had samples available for pneumococcal testing.

^d^PCV7 serotypes include 4, 6B, 9V, 14, 18C, 19F, and 23F.

^e^PCV13 serotypes include PCV7 serotypes plus 1, 3, 5, 6A, 7F, 19A, and cross-reactive serotype 6C.

^f^PCV15 serotypes include PCV13 serotypes plus 22F and 33F.

^g^PCV20 serotypes include PCV15 serotypes plus 8, 10A, 11A, 12F, 15B, and cross-reactive serotype 15C.

## DISCUSSION

Our review summarized the available data on pneumococcal serotype distribution from multiple studies that had used UAD assays among adults hospitalized with RAD + CAP. Results varied substantially by country. For example, the proportion of hospitalized RAD + CAP among older adults due to PCV20 serotypes ranged from 7% in the United States, general population, to 21% in Spain. Within a country, there was much less variation across the age groups or when including persons with underlying chronic medical conditions. Individual serotypes also varied across countries, with serotype 3 common in all European populations but not in the United States, general population; serotype 8 was highly prevalent in Spain, common in most other studies, but rare in the United States, general population; and PCV7 and PCV13 serotypes varied across countries despite long-term use of these vaccines in pediatric NIPs.

The higher-valency PCVs (PCV20 and PCV15), due to the inclusion of additional serotypes, covered a greater proportion of CAP than PCV13. Specifically, when compared to PCV13, PCV20 covered 1.3 to 2.2 times more RAD + CAP, and PCV15 covered 1.1 to 1.3 times more RAD + CAP. The detection frequency of the additional individual serotypes covered by higher valency PCVs varied by country and population. Compared with the other countries, serotype 8 was notably higher in the United States, Native American population and in Spain where it accounted for 3.1% and 7.0% of RAD + CAP among all adults, respectively. Aside from serotype 8 and compared with the other individual additional serotypes covered by higher valency PCVs, serotypes 11A and 12F were also frequently detected in some populations such as in Sweden and the US Native American population, respectively. These serotypes (8, 11A, and 12F) have also been identified among adults with invasive pneumococcal disease (IPD) and as having increased disease severity and antibiotic resistance [17, 18]. Invasive pneumococcal disease due to serotypes 8 and 12F has an upwardly increasing trend in adults of countries included or nearby to those included in this analysis [19–21]; this pattern may also be occurring in adult RAD + CAP. Although the use of PCV20 or PCV15 in adults could help further reduce vaccine-preventable disease burden among these populations, the actual benefit of higher valency PCVs will depend on a combination of factors, such as vaccine uptake, the prevalence and virulence of specific serotypes, and vaccine effectiveness (VE) against RAD + CAP caused by specific serotypes.

The ongoing occurrence of PCV13 serotype RAD + CAP among adults highlights the limitations in relying upon the indirect effects pediatric PCV programs, even when pediatric population coverage of PCV13 is high (>90% in most included countries) (Supplementary Table 2). Furthermore, most countries had longstanding PPSV23 recommendations, and some had PCV13 recommendations, but adult population coverage of vaccines was low to moderate for most countries. Finally, as noted previously, study countries used different PCV schedules and types. Because of these differences, we cannot use the current evaluation to inform why country-to-country variation in PCV13 serotype frequencies were present. Possibilities include the following: substantial PCV13 adult coverage differences in the small geographic areas covered by our studies versus national estimates; variations in circulating clones; different population mixing patterns that subsequently impacted pneumococcal transmission to older adults; pre-PCV differences in serotype distribution; differences in selection pressure related to antibiotic use and resistance prevalence; and differences in underlying risk factors that increase susceptibility to particular serotypes. Serotype 3 was notably higher than other PCV13 serotypes in most settings, which could be explained by limited PCV13 efficacy against serotype 3 carriage among children [22], indicating the potential for continued transmission within the community. Furthermore, evidence for protection against serotype 3 IPD in children is mixed, with the largest study conducted demonstrating statistically robust effectiveness against this serotype, although lower than for other PCV13 serotypes [23]. In contrast, consistent evidence exists for PCV13 direct protection against serotype 3 pneumonia and IPD among older adults [24, 25]. PCV20 is a direct extension of PCV13, and both PCV20 and PCV15 were licensed for adults based on clinical immunogenicity data [26, 27]; however, clinical effectiveness data do not yet exist for either vaccine.

The Pfizer UAD assay uses positivity cut-points calculated from a standard curve run on each assay plate together with positive- and negative-control urine samples [9]. This method emphasizes test specificity over sensitivity because it was designed to provide a relatively unbiased estimate of vaccine efficacy in a randomized controlled trial of Dutch adults (CAPiTA) [28]. For the CAPiTA trial, positivity cut-points were set based on 400 local controls that included individuals undergoing elective surgery, healthy patients with stable chronic obstructive pulmonary disease, and healthy donors with no apparent signs of pneumococcal disease [8, 9]. For all subsequent studies, 400 local controls also were collected; however, these were used only to adjust cut-points upwards from the CAPiTA cut-points, never lower. Consequently, the percentages of all RAD + CAP due to UAD serotypes should be considered a conservative estimate. This is illustrated in the United States, general population, where 4.2% of CAP in older adults was due to PCV13 serotypes [29]. Applying the VE of 45% from CAPiTA to this value would predict an approximate 1.9% reduction in all-cause CAP among PCV13 vaccinated adults. However, 3 US studies found reductions of 6.7%, 8.8%, and 10.0%, approximately 3- to 5-fold greater than predicted based on UAD results [30, 31]. Although consistent results have been demonstrated by the 3 US studies, these studies are observational studies and are subject to unmeasured confounders that may affect the VE estimates. It should also be noted that other UAD assays have been developed [7, 10]. The methods for setting positivity cut-points that have been described for these assays are different and less conservative than the Pfizer method [7, 10], likely leading to higher test sensitivity and consequently higher detection rates for serotypes included in the other assays. Comparisons across studies using different UAD technologies should therefore be performed with caution.

Use of the urine-based assays enhanced *S pneumoniae* detection, although the fold increase in detection varied across countries. When *S pneumoniae* detection by either BinaxNow or UAD assays was compared to culture, the magnitude of the increase was dependent upon the baseline culture positivity and the standard-of-care sampling rate. For example, in Greece, the *S pneumoniae* culture positivity was very low (<1% vs 5% in United States, Native Americans, and Sweden), and the standard-of-care specimen culture rate was low (68.1% vs >90% in Sweden and United States, general population). Therefore, the magnitude of the increase was highest in this setting (28 times higher for BinaxNow; 54 times higher for UAD vs 2.7 to 3.3 times higher for BinaxNow and 3.9 to 4.0 times higher for UAD in other countries). The BinaxNow and UAD assays also uniquely detected *S pneumoniae* not detected by either culture or the other urine-based assay, increasing detection by 1.2 to 1.3 times with BinaxNow and by 1.4 to 2.3 times for the UAD assays. However, even with the use of the BinaxNow and UAD assays, *S pneumoniae* prevalence may have been underestimated, although the extent of the underestimation remains unknown. Factors contributing to underestimation include a limited number of serotypes detected by UAD assays and potential lower assay sensitivity of both UAD and BinaxNow for nonbacteremic pneumonia compared with bacteremic pneumonia [9, 32]. The potential underestimation of *S pneumoniae* is supported by PCV13 VE analyses, which consistently showed a higher proportion of CAP that may be prevented by PCV13 than that detected with the UAD1 assay [30, 31, 33]. Furthermore, a reanalysis of the CAPiTA trial using a vaccine probe approach yielded an etiologic fraction of PCV13-type CAP that was higher when based on VE estimates than when based on the UAD1 assay alone [24].

Our review has limitations. First, despite the benefits of urine-based antigen detection assays, the UAD assays detect only 24 of the more than 100 known pneumococcal serotypes, all of which are vaccine serotypes, and they do not distinguish detection of certain serotypes including 6A/6C and 15B/15C. In addition, the UAD assays are only presently available for use in a research setting, which may limit generalizability of study results. Second, the sensitivity and specificity of UAD assays for nonbacteremic CAP has not been determined; therefore, findings from our study may not reflect the actual contribution of *S pneumoniae* for this syndrome. Third, although per-protocol urine collection was performed for all study participants, standard-of-care specimen collection was less complete for some studies. Therefore, comparing detection rates of *S pneumoniae* across these specimen types may overestimate the contributions of urine-based assays. Fourth, study characteristics such as the inclusion/exclusion criteria might limit generalizability beyond hospitalized adults (eg, to outpatient CAP), and selection bias might be responsible for skewed participant characteristics that would limit generalizability to the general public. Fifth, differences in data availability and variability in data collection by country limit comparisons across countries. Finally, country characteristics such as pediatric and adult PCV use could limit generalizability of results to other countries.

## CONCLUSIONS

Our data have implications for public health policy. The PCV13 serotypes remain a frequent cause of adult CAP, even in the context of mature pediatric PCV13 programs and pneumococcal vaccine recommendations in adults. Although serotype 3 was the most commonly identified PCV13 serotype, all PCV13 serotypes were represented among adult pneumonia cases. After introduction of PCVs into pediatric NIPs, substantial disease reductions were observed, not only in children but also in unvaccinated populations through herd protection. However, residual vaccine-type disease remains among adults, indicating that pediatric PCV programs by themselves are insufficient to address the full burden of adult disease. The additional serotypes included in higher valency PCVs (ie, those in PCV15 and PCV20 beyond PCV13) were also identified as important causes of adult CAP, in some studies causing over 20% of all hospitalized RAD + CAP cases among adults. These combined data suggest that directly vaccinating adults with higher valency vaccines is likely to address additional RAD + CAP cases among adults. Studies assessing PCV20 effectiveness against CAP in the context of adult higher valency PCV recommendations would inform the actual attributable fraction of disease that could be prevented. With the introduction of higher valency PCVs into the pediatric and adult immunization programs, future studies will be needed to evaluate changes in adult CAP serotype distribution. Developing UAD assays that include other serotypes would extend our understanding of CAP serotype distribution, and continued assessment of serotype distribution in adults is warranted to inform future vaccine development.

## Supplementary Data

Supplementary materials are available at *The Journal of Infectious Diseases* online. Consisting of data provided by the authors to benefit the reader, the posted materials are not copyedited and are the sole responsibility of the authors, so questions or comments should be addressed to the corresponding author.

## Supplementary Material

jiad379_Supplementary_Data

## Notes

***Acknowledgments.*** We thank Dr. Christina Bahrs for providing participant characteristics and serotype information associated with the pneumonia surveillance study conducted in Germany.

***Author contributions.*** LRG, EB, CT, MWP, LJ, and BDG contributed to study design and concept; LRG, EB, CT, CH-M, QY, MWP, LJ, and BDG contributed to data analysis and interpretation; LRG and QY wrote the first draft of the manuscript; all authors provided critical review of the manuscript; all authors have read and approved the final manuscript.

***Financial support*.** This study was sponsored by Pfizer Inc.
