# Supplementary material for: Multicountry Review of Streptococcus pneumoniae Serotype Distribution Among Adults With Community-Acquired Pneumonia
Source: J Infect Dis. 2023 Sep 4;229(1):282–93. doi: 10.1093/infdis/jiad379 (PMC10786249; doi:10.1093/infdis/jiad379)
Supplement: jiad379_Supplementary_Data [file jiad379_supplementary_data.docx]

**Supplemental Table 1. Inclusion criteria for signs and symptoms of pneumonia and exclusion criteria**

|  | **Country** | | | | | |
| --- | --- | --- | --- | --- | --- | --- |
|  | **United States**  **(General population)** | **United States**  **(Native American population)** | **Germany** | **Sweden** | **Spain** | **Greece** |
| **Inclusion criteria** |  |  |  |  |  |  |
| Number of signs or symptoms of pneumonia required for inclusion | ≥2 | ≥2 | ≥1 | ≥2 | NR ^a^ | ≥2 |
| Presence of signs or symptoms |  |  |  |  |  |  |
| Fever (temperature >38°C) within 24 hours before enrolment | ✓ | ✓ | ✓ | ✓ |  | ✓ |
| Hypothermia (<35.5°C measured by a healthcare provider) within 24 hours before enrolment | ✓ |  |  | ✓ |  | ✓ |
| Chills or rigors | ✓ |  |  | ✓ |  | ✓ |
| Pleuritic chest pain | ✓ | ✓ | ✓ | ✓ |  | ✓ |
| Cough | ✓ | ✓ | ✓ | ✓ |  | ✓ |
| Sputum production | ✓ | ✓ | ✓ | ✓ |  | ✓ |
| Dyspnea | ✓ | ✓ | ✓ | ✓ |  | ✓ |
| Tachypnoea (respiratory rate >20/min) | ✓ | ✓ |  | ✓ |  | ✓ |
| Malaise | ✓ |  |  | ✓ |  | ✓ |
| Abnormal auscultatory findings suggestive of pneumonia ^b^ | ✓ | ✓ | ✓ | ✓ |  | ✓ |
| Leukocytosis (>12K/uL) or leukopenia (<3K/uL) |  | ✓ |  |  |  |  |
| SaO_2_ <90% |  | ✓ |  |  |  |  |
| Respiratory Distress |  | ✓ |  |  |  |  |
| **Exclusion criteria** |  |  |  |  |  |  |
| Hospital acquired pneumonia | ✓ ^c^ | ✓ ^d^ | ✓ | ✓ ^c^ | ✓ ^e^ | ✓ ^c^ |
| Immunocompromising condition |  |  | ✓ ^f^ |  | ✓ ^g^ |  |
| Had severe chronic conditions or laboratory abnormalities that may increase the risk associated with study participation or may interfere with the interpretation of study results and, in the judgment of the investigator, would make the patient inappropriate for entry into this study. | ✓ |  |  |  |  |  |
| Abbreviations: NR = not reported; SaO_2_ = saturation of peripheral oxygen   1. While inclusion criteria for Spain required that CAP patients have acute signs and symptoms suggestive of lower respiratory tract infection, neither the number of signs and symptoms nor the specific signs and symptoms required for inclusion were not reported. 2. Rales or evidence of pulmonary consolidation [dullness on percussion, bronchial breath sounds, or egophony] 3. Subjects hospitalized for ≥48 hours or transferred to a study healthcare facility after already being hospitalized for ≥48 hours at any other in-patient facility (such as a community hospital); Hospital acquired pneumonia (e.g., develops signs and symptoms of pneumonia after being hospitalized for ≥48 hours) during the previous 30 days 4. Hospitalization in the 28 days prior for any cause (if duration of hospitalization ≥2 days) 5. History of hospitalization for at least 48 hours within the 2 weeks prior to current admission 6. Chemotherapy and/or neutropenia <1000 lL) during the last 28 days, therapy with corticosteroids >20 mg for >14 days, known HIV infection, immunosuppressive therapy after organ or bone marrow transplant 7. Active pulmonary tuberculosis, Sickle cell anemia, HIV infection/ AIDS or other immune suppression, Cancer treated currently or within < 1 year, Functional or anatomic asplenia | | | | | | |

**Supplemental Table 2. Pediatric and adult pneumococcal vaccine recommendations by country relevant to the study period**

| **Country** | **Pediatric vaccination** | | | | **Adult vaccination** | | | |
| --- | --- | --- | --- | --- | --- | --- | --- | --- |
|  | **PCV introduction into NIP** | **Dosing schedule used during study period** | **Vaccination coverage with final dose (reporting year ^a^)** | **References** | **Age/Risk group** | **PCV recommendation and uptake during study period, if available** | **PPSV recommendation and uptake during study period, if available** | **References** |
| **United States** | PCV7: 2000  (catch-up <2 years);  PCV13: 2010 (catch-up <5 years) | 3+1 | 93% (2016) | [1] | 19-64 years, immunocompromised persons; functional or anatomic asplenia; CSF leaks of cochlear implant | PCV13: 2012-2021^d^  23% in 19-64 years (2015) ^e^ | PPSV23: 1997  23% in 19-64 years (2015) ^e^ | [2, 3] |
|  |  |  |  |  | 19-64 years, immunocompetent persons with certain medical conditions | None^c^ | PPSV23: 1997  23% in 19-64 years (2015) ^e^ | [3] |
|  |  |  |  |  | ≥65 years, all persons | PCV13: 2014-2021^d^  1.2% to 32% from Sept 2014 to Oct 2016 | PPSV23: 1997 | [4] |
| **Germany** | PCV7: 2006 (no catch-up); PCV10/PCV13: 2009 (no catch-up) | 2+1  (In 2015, changed from 3+1 to 2+1) ^b^ | 82% (2016) | [1] | ≥2 years, immunocompetent persons with certain medical conditions | None | PPSV23: 1997  25.6% (2022) | [2-5] |
|  |  |  |  |  | ≥2 years, immunocompromised persons; functional or anatomic asplenia; CSF fistula or cochlear implant | PCV13: 2016  4.4% (2013-14) | PPSV23: 1997  4.4% (2013-14) | [6] |
|  |  |  |  |  | ≥60 years, all persons | None | PPSV23: 1998  24.1% in adults 60-74 years of age (2020) | [6, 7] |
| **Sweden** | PCV7: 2009 (no catch-up); PCV10/PCV13 (depends upon county ^c^): 2010 (no catch-up) | 2+1 | 97% (2017) | [1] | ≥2 years, immunocompromised persons; functional or anatomic asplenia; immunocompetent persons with certain medical conditions | PCV13: 2013 | PPSV23: 1994 | [6, 8, 9] |
|  |  |  |  |  | ≥65 years, all persons | None | PPSV23: 1994  <30% (2015) | [9, 10] |
| **Spain (Catalonia, Valencia, Basque country, Galicia)** | PCV13: 2016 (Catalonia); 2015 (Valencia, Basque country); 2011 (Galicia) | 2+1 | 93% (2018) | [1, 11, 12] | >2 years, immunocompromised persons; immunocompetent persons with certain medical conditions | PCV13: 2012  0.2% in adults >50 years in Catalonia (2015) | PPSV23: 2000 or 2001  59.2% in adults >50 years in Catalonia (2015) | [10, 13] |
|  |  |  |  |  | ≥65 years, all persons | PCV13: 2017 (Galicia only)  35% (2017) and 23% (2018) for adults 65 years only ^f^ | PPSV23: 2000  69.7% in Catalonia (2015)  24% in Galicia (2017) | [9, 10, 13, 14] |
| **Greece** | PCV7: 2006 (catch-up <5 years); PCV10: 2009 (no catch-up); PCV13: 2010 (catch-up <5 years) | 2+1  (In 2019, changed from 3+1 to 2+1) | 96% (2018) | [1, 13, 15] | 19-64 years, immunocompromised persons; functional or anatomic asplenia; immunocompetent with certain medical conditions | PCV13: 2011  <10% in 2019 | PPSV23: 2011 | [16-19] |
|  |  |  |  |  | ≥65 years, all persons | PCV13: 2011  >30% in 2019 | PPSV23: 2011  23% in adults ≥60 years of age in 2019 | [18, 20] |
| Abbreviations: CSF = cerebrospinal fluid; NIP = national immunization program; PCV= pneumococcal conjugate vaccine; PCV7 = 7-valent pneumococcal conjugate vaccine; PCV10 = 10-valent pneumococcal conjugate vaccine; PCV13 = 13-valent pneumococcal conjugate vaccine; PCV20 = 20-valent pneumococcal conjugate vaccine; PPSV = pneumococcal polysaccharide vaccine; PPSV23 = 23-valent pneumococcal polysaccharide vaccine  a. Pediatric vaccination coverage was provided for approximately the mid-point study year using data from WHO unless reported directly in the study publication (Spain).  b. Germany is a physician’s choice market where both PCV10 and PCV13 are recommended. The market share is >90% for PCV13 [21].  c. In Sweden, choice of either PCV10 or PCV13 varies by county. In Malmo, where the adult CAP study was conducted, PCV13 was used.  d. In the United States, in 2022, PCV20 or PCV15 + PPSV23 were recommended for adults 19-64 years who were immunocompromised or with certain chronic medical conditions and for all adults 65 years and older, replacing the previous recommendation.  e. The uptake estimate among US adults 19-64 years of age refers to those who are at increased risk of pneumococcal disease and ever vaccinated with PCV13 or PPSV23. Adults were considered at increased risk for pneumococcal disease if they had ever been told by a doctor or other health professional that they had diabetes, emphysema, chronic obstructive pulmonary disease, coronary heart disease, angina, heart attack, or other heart condition; had a diagnosis of cancer during the previous 12 months (excluding nonmelanoma skin cancer); had ever been told by a doctor or other health professional that they had lymphoma, leukemia, or blood cancer; had been told by a doctor or other health professional that they had chronic bronchitis or weak or failing kidneys during the preceding 12 months; had an asthma episode or attack during the preceding 12 months; or were current smokers. For hepatitis A and hepatitis B vaccination, data were collected on selected respondent characteristics that increase the risk for infection (travel to countries where hepatitis A infections are endemic and having chronic liver disease; having diabetes, travel to countries where hepatitis B infections are endemic, and having chronic liver disease, respectively).  f. In the Galicia region of Spain, PCV13 was initially recommended only for adults turning 65 years of age. | | | | | | | | |

**Supplemental Table 3. *S pneumoniae* detection by laboratory method among participants with RAD+CAP**

|  | **United States**  **(General population)** | | | **United States**  **(Native American population)** | | | **Germany ^a^** | | | **Sweden** | | | **Spain ^b^** | | | **Greece** | | |
| --- | --- | --- | --- | --- | --- | --- | --- | --- | --- | --- | --- | --- | --- | --- | --- | --- | --- | --- |
| Age group, years | 18-64 | ≥65 | ≥18 | 18-64 | ≥65 | ≥18 | 18-59 with CMC | ≥60 | ≥18 | 18-64 | ≥65 | ≥18 | 18-64 | ≥65 | ≥18 | 19-64 | ≥65 | ≥19 |
| Total number of RAD+CAP cases | 5708 | 6347 | 12055 | 274 | 298 | 572 ^c^ | 316 | 792 | 1343 | 169 | 349 | 518 | 401 | 620 | 1021 | 154 | 328 | 482 |
| *S pneumoniae* detection by diagnostic test, n/N (%) |  |  |  |  |  |  |  |  |  |  |  |  |  |  |  |  |  |  |
| Any method | 760/5708 (13.3) | 722/6347 (11.4) | 1482/12055 (12.3) | 87/274 (31.8) | 77/298 (25.8) | 164/572 (28.6) | NR | NR | NR | 46/169 (27.2) | 80/349 (22.9) | 126/518 (24.3) | 141/401 (35.2) | 206/620 (33.2) | 347/1021 (34.0) | 25/154 (16.2) | 40/328 (12.2) | 65/482 (13.5) |
| Culture | 143/5389 (2.7) | 119/5993  (2.0) | 262/11382 (2.3) | 19/274 (6.9) | 10/298 (3.4) | 29/483 (6.0) | NR | NR | NR | 11/166 (6.6) | 13/312 (4.2) | 24/478 (5.0) | NR | NR | NR | 1/154 (0.6) | 0 | 1/482 (0.2) |
| UAD | 548/5707 (9.6) | 480/6347 (7.6) | 1028/12054 (8.5) | 71/274 (25.9) | 49/298 (16.4) | 120/572 (21.0) | 38/316 (12.1) | 113/792 (14.4) | 183/1343 (13.7) | 38/169 (22.5) | 59/349 (16.9) | 97/518 (18.7) | NR | NR | NR | 22/154  (14.3) | 32/328  (9.8) | 54/482 (11.2) |
| BinaxNOW™ | 346/5708 (6.1) | 353/6347 (5.6) | 699/12055 (5.8) | 52/274 (19.0) | 50/298 (16.8) | 102/572 (17.8) | NR | NR | NR | 25/169 (14.8) | 43/349 (12.3) | 68/518 (13.1) | NR | NR | 523/3107 (16.8) ^d^ | 7/154  (4.5) | 21/328  (6.4) | 28/482 (5.8) |
|  |  |  |  |  |  |  |  |  |  |  |  |  |  |  |  |  |  |  |
| *S pneumoniae* detection by groups of diagnostic methods (N) | 760 | 722 | 1482 | NR | NR | 143 ^e^ | NR | NR | NR | 46 | 80 | 126 | 141 | 206 | 347 | 25 | 40 | 65 |
| 1: UAD alone, n/N (%) | 330/760 (43.4) | 303/722 (42.0) | 633/1482 (42.7) | NR | NR | 44/143 (30.7) | NR | NR | NR | 17/46 (37.0) | 31/80 (38.8) | 48/126 (38.1) | NR | NR | NR | 18/25 (72.0) | 19/40 (47.5) | 37/65 (56.9) |
| 2: UAD + BinaxNOW™, n/N (%) | 131/760 (17.2) | 108/722 (15.0) | 239/1482 (16.1) | NR | NR | 40/143 (27.9) | NR | NR | NR | 11/46 (23.9) | 16/80 (20.0) | 27/126 (21.4) | NR | NR | NR | 4/25 (16.0) | 13/40 (32.5) | 17/65 (26.2) |
| 3: BinaxNOW™ alone, n/N (%) | 156/760 (20.5) | 192/722 (26.6) | 348/1482 (23.5) | NR | NR | 32/143 (22.4) | NR | NR | NR | 7/46 (15.2) | 20/80 (25.0) | 27/126 (21.4) | NR | NR | NR | 2/25 (8.0) | 8/40 (20.0) | 10/65 (15.4) |
| 4: BinaxNOW™ + Culture, n/N (%) | 24/760 (3.2) | 14/722 (1.9) | 38/1482 (2.6) | NR | NR | 2/143 (1.4) | NR | NR | NR | 0/46 (0.0) | 0/80 (0.0) | 0/126 (0) | NR | NR | NR | 1/25 (4.0) | 0 | 1/65 (1.5) |
| 5: UAD + Culture, n/N (%) | 52/760 (6.8) | 30/722 (4.2) | 82/1482 (5.5) | NR | NR | 8/143 (5.6) | NR | NR | NR | 3/46 (6.5) | 5/80 (6.3) | 8/126 (6.3) | NR | NR | NR | 0 | 0 | 0 |
| 6: Culture alone, n/N (%) | 32/760 (4.2) | 36/722 (5.0) | 68/1482 (4.6) | NR | NR | 1/143 (0.7) | NR | NR | NR | 1/46 (2.2) | 1/80 (1.3) | 2/126 (1.6) | NR | NR | NR | 0 | 0 | 0 |
| 7: UAD + BinaxNOW™ + Culture, n/N (%) | 35/760 (4.6) | 39/722 (5.4) | 74/1482 (5.0) | NR | NR | 16/143 (11.2) | NR | NR | NR | 7/46 (15.2) | 7/80 (8.8) | 14/126 (11.1) | NR | NR | NR | 0 | 0 | 0 |
|  |  |  |  |  |  |  |  |  |  |  |  |  |  |  |  |  |  |  |
| Fold change calculations, n/N (change) |  |  |  |  |  |  |  |  |  |  |  |  |  |  |  |  |  |  |
| Unique contribution of UAD assays (Figure 1A) ^f^ | 760/430 (1.8) | 722/419 (1.7) | 1482/849 (1.8) | -- | -- | 143/99 (1.4) | -- | -- | -- | 46/29 (1.6) | 80/49 (1.6) | 126/78 (1.6) | -- | -- | -- | 25/7 (3.8) | 40/21 (1.9) | 65/28 (2.3) |
| Unique contribution of BinaxNOW™  (Figure 1B) ^g^ | 760/604 (1.3) | 722/530 (1.4) | 1482/1134 (1.3) | -- | -- | 143/111 (1.3) | -- | -- | -- | 46/39 (1.2) | 80/60 (1.3) | 126/99 (1.3) | -- | -- | -- | 25/23 (1.1) | 40/32 (1.3) | 65/55 (1.2) |
| Contribution of UAD vs. culture (Figure 1C) ^h^ | 548/143 (3.8) | 480/119 (4.0) | 1028/262 (3.9) | -- | -- | 108/27 (4.0) | -- | -- | -- | 38/11 (3.5) | 59/13 (4.5) | 97/24 (4.0) | -- | -- | -- | 22/1 (22.0) | 32/0 (UND) | 54/1 (54.0) |
| Contribution of BinaxNOW™ test vs. culture (Figure 1C) ^i^ | 346/143 (2.4) | 353/119 (3.0) | 699/262 (2.7) | -- | -- | 90/27 (3.3) | -- | -- | -- | 25/11 (2.3) | 43/13 (3.3) | 68/24 (2.8) | -- | -- | -- | 7/1 (7.0) | 21/0 (UND) | 28/1 (28.) |
| Abbreviations: CMC = chronic medical conditions, n = numerator count, N = denominator count, NR = not reported, RAD+CAP = radiologically confirmed community-acquired pneumonia, UAD = urinary antigen detection, UND = undefined  a. Germany did not include results of standard of care specimens cultured for *S. pneumoniae* nor was the BinaxNOW™ test run on urine samples.  b. Unless otherwise noted, data from Spain were restricted to the period of November 2016 – November 2018 when UAD1 and UAD2 assays were both used.  c. A total of 580 patients were included in the RAD+CAP population; of these, 572 had samples available for pneumococcal testing.  d.BinaxNOW™ test results from Spain were reported for the entire study period of November 2011 – November 2018.  e. The number 143 represented the number of patients with a positive *S. pneumoniae* test result by any test among those with all three tests performed.  f. Unique contribution of UAD assays calculated as the number of *S. pneumoniae* samples detected by UAD + culture + BinaxNOW™ divided by the number of *S. pneumoniae* samples detected by culture + BinaxNOW™  g. Unique contribution of BinaxNOW™ assays calculated as number of *S. pneumoniae* samples detected by UAD + culture + BinaxNOW™ divided by the number of *S. pneumoniae* samples detected by culture + UAD  h. Contribution of UAD vs. culture calculated as the number of *S. pneumoniae* samples detected by UAD assays divided by the number of *S. pneumoniae* samples detected by culture  i. Contribution of BinaxNOW™ vs. culture calculated as the number of *S. pneumoniae* samples detected by BinaxNOW™ divided by the number of *S. pneumoniae* samples detected by culture. | | | | | | | | | | | | | | | | | | |

**Supplemental Table 4. Age-stratified serotype distribution as detected by culture and UAD assays among participants with RAD+CAP**

|  | **United States (General population)** | | | **United States (Native American population) ^a^** | | | **Germany** | | | **Sweden** | | | **Spain ^a,b^** | | | **Greece ^a^** | | |
| --- | --- | --- | --- | --- | --- | --- | --- | --- | --- | --- | --- | --- | --- | --- | --- | --- | --- | --- |
| Age group, years | 18-64 | ≥65 | ≥18 | 18-64 | ≥65 | ≥18 | 18-59 with CMC | ≥60 | ≥18 | 18-64 | ≥65 | ≥18 | 18-64 | ≥65 | ≥18 | 19-64 | ≥65 | ≥19 |
| Total number of RAD+CAP cases | 5708 | 6347 | 12055 | 274 | 298 | 572 ^c^ | 316 | 792 | 1343 | 169 | 349 | 518 | 401 | 620 | 1021 | 154 | 328 | 482 |
| Individual serotypes, n (%) |  |  |  |  |  |  |  |  |  |  |  |  |  |  |  |  |  |  |
| 4 | 4 (0.1) | 8 (0.1) | 12 (1.0) | *0* | *1 (0.3)* | *1 (0.2)* | 4 (1.3) | 3 (0.4) | 9 (0.7) | 0 | 3 (0.9) | 3 (0.6) | *1 (0.2)* | *1 (0.2)* | *2 (0.2)* | 1 (0.6) | 0 | 1 (0.2) |
| 6B | 4 (0.1) | 2 (0.0) | 6 (0.1) | *0* | *0* | *0* | 0 | 1 (0.1) | 1 (0.1) | 0 | 0 | 0 (0) | *0* | *1 (0.2)* | *1 (0.1)* | 0 | 0 | 0 |
| 9V | 10 (0.2) | 5 (0.1) | 15 (0.1) | *0* | *0* | *0* | 0 | 2 (0.3) | 3 (0.2) | 0 | 0 | 0 (0) | *1 (0.2)* | *0* | *1 (0.1)* | 0 | 1 (0.3) | 1 (0.2) |
| 14 | 15 (0.3) | 11 (0.2) | 26 (0.2) | 1 (0.4) | 2 (0.7) | 3 (0.6) | 1 (0.3) | 1 (0.1) | 3 (0.2) | 0 | 2 (0.6) | 2 (0.4) | 6 (1.5) | 5 (0.8) | 11 (1.1) | 0 | 0 | 0 |
| 18C | 16 (0.3) | 6 (0.1) | 22 (0.2) | 1 (0.4) | 2 (0.7) | 3 (0.6) | 1 (0.3) | 2 (0.3) | 4 (0.3) | 1 (0.6) | 1 (0.3) | 2 (0.4) | 0 | 1 (0.2) | 1 (0.1) | 0 | 0 | 0 |
| 19F | 9 (0.2) | 10 (0.2) | 19 (0.2) | 0 | 0 | 0 | 2 (0.6) | 2 (0.3) | 5 (0.4) | 0 | 1 (0.3) | 1 (0.2) | 1 (0.2) | 3 (0.5) | 4 (0.4) | 0 | 0 | 0 |
| 23F | 17 (0.3) | 12 (0.2) | 29 (0.2) | 0 | 0 | 0 | 0 | 5 (0.6) | 5 (0.4) | 1 (0.6) | 0 | 1 (0.2) | 2 (0.5) | 3 (0.5) | 5 (0.5) | 1 (0.6) | 3 (0.9) | 4 (0.8) |
| 1 | 14 (0.3) | 15 (0.2) | 29 (0.2) | 0 | 0 | 0 | 1 (0.3) | 1 (0.1) | 2 (0.1) | 0 | 0 | 0 | 1 (0.2) | 0 | 1 (0.1) | 0 | 1 (0.3) | 1 (0.2) |
| 3 | 73 (1.3) | 57 (0.9) | 130 (1.1) | 17 (6.2) | 18 (6.0) | 35 (6.1) | 10 (3.2) | 33 (4.2) | 49 (3.7) | 9 (5.3) | 17 (4.9) | 26 (5) | 29 (7.2) | 56 (9.0) | 85 (8.3) | 11 (7.1) | 9 (2.7) | 20 (4.1) |
| 5 | 42 (0.7) | 36 (0.6) | 78 (0.7) | 0 | 0 | 0 | 0 | 1 (0.1) | 1 (0.1) | 3 (1.8) | 5 (1.4) | 8 (1.5) | 1 (0.2) | 1 (0.2) | 2 (0.2) | 0 | 1 (0.3) | 1 (0.2) |
| 6A/6C | 21 (0.4) | 33 (0.5) | 54 (0.5) | 2 (0.7) | 3 (1.0) | 5 (0.8) | 1 (0.3) | 4 (0.5) | 6 (0.4) | 0 | 3 (0.9) | 3 (0.6) | 1 (0.2) | 4 (0.6) | 5 (0.5) | 1 (0.6) | 1 (0.3) | 2 (0.4) |
| 7F | 30 (0.5) | 24 (0.4) | 54 (0.5) | 1 (0.4) | 1 (0.3) | 2 (0.3) | 0 | 5 (0.6) | 9 (0.7) | 2 (1.2) | 0 | 2 (0.4) | 3 (0.7) | 2 (0.3) | 5 (0.5) | 0 | 0 | 0 |
| 19A | 79 (1.4) | 73 (1.2) | 152 (1.3) | 2 (0.7) | 1 (0.3) | 3 (0.5) | 3 (0.9) | 3 (0.4) | 8 (0.6) | 6 (3.6) | 4 (1.1) | 10 (1.9) | 5 (1.2) | 6 (1.0) | 11 (1.1) | 2 (1.3) | 4 (1.2) | 6 (1.2) |
| 8 | 25 (0.4) | 11 (0.2) | 36 (0.3) | *13 (4.7)* | *5 (1.7)* | *18 (3.1)* | 7 (2.2) | 9 (1.1) | 21 (1.6) | 7 (4.1) | 3 (0.3) | 10 (1.9) | 46 (11.5) | 25 (4.0) | 71 (7.0) | 0 | 3 (0.9) | 3 (0.6) |
| 10A | 27 (0.5) | 24 (0.4) | 51 (0.4) | *2 (0.7)* | *0* | *2 (0.3)* | 1 (0.3) | 3 (0.4) | 6 (0.4) | 0 | 1 (0.3) | 1 (0.2) | *1 (0.2)* | *4 (0.6)* | *5 (0.5)* | 1 (0.6) | 1 (0.3) | 2 (0.4) |
| 11A | 51 (0.9) | 46 (0.7) | 97 (0.8) | *0* | *4 (1.3)* | *4 (0.7)* | 1 (0.3) | 10 (1.3) | 11 (0.8) | 4 (2.4) | 6 (1.7) | 10 (1.9) | *3 (0.7)* | *5 (0.8)* | *8 (0.8)* | 1 (0.6) | 1 (0.3) | 2 (0.4) |
| 12F | 21 (0.4) | 13 (0.2) | 34 (0.3) | *9 (3.3)* | *0* | *9 (1.6)* | 1 (0.3) | 1 (0.1) | 3 (0.2) | 0 | 1 (0.3) | 1 (0.2) | 8 (2.0) | 2 (0.3) | *10 (1.0)* | 0 | 0 | 0 |
| 15B/15C | 15 (0.3) | 7 (0.1) | 22 (0.2) | *0* | *0* | *0* | 0 | 3 (0.4) | 3 (0.2) | 2 (1.2) | 0 | 2 (0.4) | *0* | *1 (0.2)* | *1 (0.1)* | 0 | 1 (0.3) | 1 (0.2) |
| 22F | 67 (1.2) | 59 (0.9) | 126 (1.1) | *3 (1.1)* | *3 (1.0)* | *6 (1.1)* | 3 (1.0) | 9 (1.1) | 13 (1.0) | 0 | 6 (1.7) | 6 (1.2) | *3 (0.7)* | 9 (1.5) | 12 (1.2) | 2 (1.3) | 1 (0.3) | 3 (0.6) |
| 33F | 16 (0.3) | 24 (0.4) | 40 (0.3) | *3 (1.1)* | *1 (0.3)* | *4 (0.7)* | 2 (0.6) | 5 (0.6) | 8 (0.6) | 2 (1.2) | 1 (0.3) | 3 (0.6) | *0* | *4 (0.6)* | *4 (0.4)* | 0 | 1 (0.3) | 1 (0.2) |
| 2 | 0 | 3 (0.1) | 3 (0.0) | *0* | *0* | *0* | 0 | 1 (0.1) | 1 (0.1) | 0 | 0 | 0 | *0* | *0* | *0* | 0 | 1 (0.3) | 1 (0.2) |
| 9N | 37 (0.7) | 23 (0.4) | 60 (0.5) | *3 (1.1)* | *1 (0.3)* | *4 (0.7)* | 0 | 8 (1.0) | 11 (0.8) | 3 (1.8) | 4 (1.1) | 7 (1.4) | 7 (1.7) | 8 (1.3) | 15 (1.5) | 2 (1.3) | 2 (0.6) | 4 (0.8) |
| 17F | 15 (0.3) | 15 (0.2) | 30 (0.3) | *2 (0.7)* | *5 (1.7)* | *7 (1.2)* | 0 | 3 (0.4) | 4 (0.3) | 1 (0.6) | 3 (0.9) | 4 (0.8) | *1 (0.2)* | *6 (1.0)* | *7 (0.7)* | 1 (0.6) | 1 (0.3) | 2 (0.4) |
| 20 | 17 (0.3) | 14 (0.2) | 31 (0.3) | *12 (7.3)* | *3 (1.0)* | *15 (2.6)* | 0 | 3 (0.4) | 3 (0.2) | 0 | 0 | 0 | *0* | *1 (0.2)* | *1 (0.1)* | 1 (0.6) | 3 (0.9) | 4 (0.8) |
| Note: Serotype distribution data separately for bacteremic and nonbacteremic cases were not necessarily available in the original publications.  Abbreviations: CAP = community acquired pneumonia, CMC = chronic medical conditions  a. Serotype counts in italics from US (Native American population), Spain, and Greece were estimated using WebPlotDigitizer, a web-based tool used to extract numerical data from figures.  b. Serotype counts represent data between November 2016 – November 2018 only.  c. A total of 580 patients were included in the RAD+CAP population; of these, 572 had samples available for pneumococcal testing. | | | | | | | | | | | | | | | | | | |

**Supplemental Table 5. Distribution of serotypes detected by culture only among participants with RAD+CAP ^a^**

|  | **United States**  **(General population)** | | | **United States**  **(Native American population) ^b^** | | | **Sweden** | | | **Spain ^b,c^** | | |
| --- | --- | --- | --- | --- | --- | --- | --- | --- | --- | --- | --- | --- |
| Age group, years | ≥18 | 18-64 | ≥65 | ≥18 | 18-64 | ≥65 | ≥18 | 18-64 | ≥65 | ≥18 | 18-64 | ≥65 |
| Total number of RAD+CAP cases | 12055 | 5708 | 6347 | 572 ^d^ | 274 | 298 | 518 | 169 | 349 | 1021 | 401 | 620 |
| Total *S. pneumoniae* positive by culture | 262/11382 (2.3) | 143/5389 (2.7) | 119/5993  (2.0) | 29/572 (5.1) | 19/274 (6.9) | 10/298 (3.4) | 24/478 (5.0) | 11/166 (6.6) | 13/312 (4.2) | NR | NR | NR |
|  |  |  |  |  |  |  |  |  |  |  |  |  |
| Non-UAD serotypes detected by culture  only, n (%) | 64 (0.5) | 37 (0.6) | 27 (0.4) | *4 (0.7)* | 1 (0.4) | 3 (1.0) | 3 (0.6) | 1 (0.6) | 2 (0.6) | *3 (0.3)* | *0* | *3 (0.5)* |
| 7B | 1 (0.0) | 1 (0.0) | 0 | *0* | *0* | *0* | 0 | 0 | 0 | *0* | *0* | *0* |
| 7C | 3 (0.0) | 3 (0.0) | 0 | *0* | *0* | *0* | 0 | 0 | 0 | *1 (0.1)* | *0* | *1 (0.2)* |
| 11C | 1 (0.0) | 1 (0.0) | 0 | *0* | *0* | *0* | 0 | 0 | 0 | *0* | *0* | *0* |
| 12 ^e^ | 0 | 0 | 0 | *0* | *0* | *0* | 1 (0.2) | 0 | 1 (0.3) | *0* | *0* | *0* |
| 13 | 1 (0.0) | 0 | 1 (0.0) | *0* | *0* | *0* | 0 | 0 | 0 | *0* | *0* | *0* |
| 15A | 9 (0.0) | 7 (0.1) | 2 (0.0) | *0* | *0* | *0* | 0 | 0 | 0 | *0* | *0* | *0* |
| 16F | 13 (0.1) | 7 (0.1) | 6 (0.1) | *0* | *0* | *0* | 0 | 0 | 0 | *0* | *0* | *0* |
| 21 | 6 (0.0) | 3 (0.0) | 3 (0.0) | *0* | *0* | *0* | 0 | 0 | 0 | *0* | *0* | *0* |
| 23A | 2 (0.0) | 1 (0.0) | 1 (0.0) | *0* | *0* | *0* | 0 | 0 | 0 | *0* | *0* | *0* |
| 23B | 7 (0.0) | 3 (0.0) | 4 (0.0) | *0* | *0* | *0* | 0 | 0 | 0 | *0* | *0* | *0* |
| 25F | 1 (0.0) | 1 (0.0) | 0 | *0* | *0* | *0* | 0 | 0 | 0 | *0* | *0* | *0* |
| 31 | 3 (0.0) | 1 (0.0) | 2 (0.0) | *1 (0.2)* | *0* | *1 (0.3)* | 0 | 0 | 0 | *1 (0.1)* | *0* | *1 (0.2)* |
| 34 | 6 (0.0) | 4 (0.0) | 2 (0.0) | *0* | *0* | *0* | 0 | 0 | 0 | *0* | *0* | *0* |
| 35B | 9 (0.0) | 4 (0.0) | 5 (0.0) | *2 (0.4)* | *1 (0.4)* | *1 (0.3)* | 1 (0.2) | 0 | 1 (0.3) | *1 (0.1)* | *0* | *1 (0.2)* |
| 37 | 0 | 0 | 0 | *1 (0.2)* | *0* | *1 (0.3)* | 1 (0.2) | 1 (0.6) | 0 | *0* | *0* | *0* |
| 38 | 2 (0.0) | 1 (0.0) | 1 (0.0) | *0* | *0* | *0* | 0 | 0 | 0 | *0* | *0* | *0* |
| Abbreviations: NR = not reported; RAD+CAP = radiologically confirmed community acquired pneumonia; UAD = urinary antigen detection assay  a. Greece did not report any non-UAD serotypes detected by culture only, and Germany serotype distribution was based on UAD1/UAD2 results only.  b. Serotype counts in italics from US (Native American population), Spain, and Greece were estimated using WebPlotDigitizer, a web-based tool used to extract numerical data from figures.  c. Serotype counts represent data between November 2016 – November 2018 only.  d. A total of 580 patients were included in the RAD+CAP population; of these, 572 had samples available for pneumococcal testing.  e. The serotype of the cultured serogroup 12 isolate was not identified. | | | | | | | | | | | | |

**References**

1. WHO. Pneumococcal vaccination coverage. Available at: <https://immunizationdata.who.int/pages/coverage/pcv.html>. Accessed October 28, 2022.

2. Prevention of pneumococcal disease: recommendations of the Advisory Committee on Immunization Practices (ACIP). MMWR Recomm Rep **1997**; 46:1-24.

3. Williams WW, Lu PJ, O'Halloran A, et al. Surveillance of Vaccination Coverage among Adult Populations - United States, 2015. MMWR Surveill Summ **2017**; 66:1-28.

4. McLaughlin JM, Swerdlow DL, Khan F, et al. Disparities in uptake of 13-valent pneumococcal conjugate vaccine among older adults in the United States. Hum Vaccin Immunother **2019**; 15:841-9.

5. Robert Koch Institut. Empfehlungen der Ständigen Impfkommission (STIKO) am Robert Koch Institut 2018-2019. Available at: <https://edoc.rki.de/bitstream/handle/176904/5715.4/34_2018_Korrektur_V3.pdf?sequence=6&isAllowed=y>. Accessed March 22, 2022.

6. Falkenhorst G, Remschmidt C, Harder T, et al. Background paper to the updated pneumococcal vaccination recommendation for older adults in Germany. Bundesgesundheitsblatt Gesundheitsforschung Gesundheitsschutz **2016**; 59:1623-57.

7. Richtlinie des Gemeinsamen Bundesausschusses über Schutzimpfungen nach § 20i Abs. 1 SGB V. June 29, 2018.

8. Robert Koch Institut. Wissenschaftliche Begründung zur Änderung der Pneumokokken-Impfempfehlung für Säuglinge. Epidemiol Bull. 2015;(36):377–92.

9. Edhag O, Tüll P. Vaccination mot pneumokocker (Swedish). Recommendations from the Swedish National Board of Health and Welfare, 1994(26):1–4.

10. Naucler P, Henriques-Normark B, Hedlund J, Galanis I, Granath F, Ortqvist A. The changing epidemiology of community-acquired pneumonia: nationwide register-based study in Sweden. J Intern Med **2019**; 286:689-701.

11. Robert-Koch-Institut. Impfquoten bei Erwachsenen in Deutschland. Epidemiologisches Bulletin. 2020(47):2.

12. Public Health Agency of Sweden. Folkhälsomyndigheten. Rekommendationer om pneumokockvaccination till riskgrupper. 2020.

13. Vila-Corcoles A, Ochoa-Gondar O, Hospital I, et al. Pneumococcal vaccination coverages among low-, intermediate-, and high-risk adults in Catalonia. Hum Vaccin Immunother **2016**; 12:2953-8.

14. Janusinfo Stockholms Läns Landsting. Konjugatvaccin i kombination med polysackaridvaccin för vaccination av barn från 2 års ålder och vuxna med stor risk för att drabbas av pneumokockinfektion. 2013. Available at: <http://www.janusinfo.se/Behandling/Expertradsutlatanden/Vaccinationer/Konjugatvaccin-for-vaccination-av-barn-aldre-an-5-ar-och-vuxna-med-stor-risk-for-att-drabbas-av-pneumokockinfektion>. Accessed March 20, 2022.

15. Ministerio de Sanidad, Servicios Sociales e Igualdad. Vacunación frente a neumococo en grupos de riesgo. May 2015. Available at: <https://www.mscbs.gob.es/profesionales/saludPublica/prevPromocion/vacunaciones/docs/Neumococo_Gruposriesgo.pdf>. Accessed October 28, 2022.

16. Ponencia de Programas y Registro de Vacunaciones. Revisión del Calendario de Vacunación. Comisión de Salud Pública del Consejo Interterritorial del Sistema Nacional de Salud. Ministerio de Sanidad, Servicios Sociales e Igualdad, 2016. Available at: <https://www.mscbs.gob.es/profesionales/saludPublica/prevPromocion/vacunaciones/docs/Revision_CalendarioVacunacion.pdf>. Accessed March 20, 2022.

17. Castiglia P. Recommendations for pneumococcal immunization outside routine childhood immunization programs in Western Europe. Adv Ther **2014**; 31:1011-44.

18. Liapikou A, Konstantinidis A, Kossyvaki V, et al. Pneumococcal serotypes in adults hospitalized with community-acquired pneumonia in Greece using urinary antigen detection tests: the EGNATIA study, November 2017 - April 2019. Hum Vaccin Immunother **2022**; 18:2079923.

19. Xirogianni A, Marmaras N, Georgakopoulou T, et al. Pneumococcal meningitis in Greece: A retrospective serotype surveillance study in the post-PCV13 era (2010-2020). Vaccine **2022**; 40:5079-87.

20. Papagiannis D, Rachiotis G, Mariolis A, Zafiriou E, Gourgoulianis KI. Vaccination Coverage of the Elderly in Greece: A Cross-Sectional Nationwide Study. Can J Infect Dis Med Microbiol **2020**; 2020:5459793.

21. Perniciaro S, Imohl M, Fitzner C, van der Linden M. Regional variations in serotype distribution and vaccination status in children under six years of age with invasive pneumococcal disease in Germany. PLoS One **2019**; 14:e0210278.
